# Microcomb-driven silicon photonic systems

**DOI:** 10.1038/s41586-022-04579-3

**Published:** 2022-05-18

**Authors:** Haowen Shu, Lin Chang, Yuansheng Tao, Bitao Shen, Weiqiang Xie, Ming Jin, Andrew Netherton, Zihan Tao, Xuguang Zhang, Ruixuan Chen, Bowen Bai, Jun Qin, Shaohua Yu, Xingjun Wang, John E. Bowers

**Affiliations:** 1 State Key Laboratory of Advanced Optical Communications System and Networks, School of Electronics, Peking University, Beijing, China; 2 Department of Electrical and Computer Engineering, University of California Santa Barbara, Santa Barbara, CA USA; 3 Peng Cheng Laboratory, Shenzhen, China; 4 Frontiers Science Center for Nano-optoelectronics, Peking University, Beijing, China

**Keywords:** Integrated optics, Silicon photonics, Frequency combs

## Abstract

Microcombs have sparked a surge of applications over the past decade, ranging from optical communications to metrology^1–4^. Despite their diverse deployment, most microcomb-based systems rely on a large amount of bulky elements and equipment to fulfil their desired functions, which is complicated, expensive and power consuming. By contrast, foundry-based silicon photonics (SiPh) has had remarkable success in providing versatile functionality in a scalable and low-cost manner^5–7^, but its available chip-based light sources lack the capacity for parallelization, which limits the scope of SiPh applications. Here we combine these two technologies by using a power-efficient and operationally simple aluminium-gallium-arsenide-on-insulator microcomb source to drive complementary metal–oxide–semiconductor SiPh engines. We present two important chip-scale photonic systems for optical data transmission and microwave photonics, respectively. A microcomb-based integrated photonic data link is demonstrated, based on a pulse-amplitude four-level modulation scheme with a two-terabit-per-second aggregate rate, and a highly reconfigurable microwave photonic filter with a high level of integration is constructed using a time-stretch approach. Such synergy of a microcomb and SiPh integrated components is an essential step towards the next generation of fully integrated photonic systems.

## Abstract (web summary)

A simple and power-efficient microcomb source is used to drive complementary metal–oxide–semiconductor silicon photonic engines, a step towards the next generation of fully integrated photonic systems.

## Main

Integrated photonics is profoundly impacting data communication and signal processing^8–10^. A crucial development in the past decade is the demonstration of Kerr microcombs, which provide mutually coherent and equidistant optical frequency lines generated by microresonators^1,11,12^. With a wide range of microcomb-based optoelectronic systems^2,4,13–18^ demonstrated recently, these integrated light sources hold the promise to extend the application space of integrated photonics to a much broader scope. However, despite the tremendous progress made in microcomb integration^19–23^, in almost all system-level demonstrations leveraging microcomb technologies, the passive comb generators are still the only integrated component. The rest of the system, including the comb pumping lasers, passive and active optical components, and the supporting electronics, usually rely on bulky, expensive and power-consuming equipment, thereby undermining the promised benefits of integrated photonics.

In contrast, the advances in silicon photonics (SiPh) technology have provided a scalable and low-cost solution to miniaturize optical systems^6,24,25^, benefiting from complementary metal–oxide–semiconductor (CMOS)-compatible manufacturing. These ‘photonic engines’, have been commercialized in data interconnects^26,27^, and widely applied in other fields^28–31^. Yet, a key ingredient missing from foundry-based silicon-on-insulator (SOI) photonic integrated circuits (PICs) is the multiple wavelength source. For example, the current state-of-the-art photonic transceiver module contains an eight-channel distributed feedback laser (DFB) array for wavelength division multiplexing (WDM)^32^. Increasing the channel count in such a system requires considerable design effort, such as line-to-line spacing stabilization and increased assembly workload. Moreover, the lack of mutual coherence among channel lines restricts many applications, such as precise time–frequency metrology.

Although interfacing these two technologies is essential to address the aforementioned problems on both sides, until now, such a combination has remained elusive. Previously, although the combinations of a microcomb and other photonic components have shown potential in optical computation^15^, atomic clocks^4^ and synthesizer systems^3^, these integrated demonstrations usually rely on specialized fabrication processes unsuitable for high-volume production. Moreover, comb start-up^33,34^ and stabilization techniques^35,36^, which require high-performance discrete optics and electronic components, markedly increase the operation complexity and system size. Recent progress in hybrid or heterogeneous laser-microcomb integration enables on-chip comb generation in a simplified manner^21–23^, but these schemes add complexity in processing. These difficulties, along with the extra expenditures on multi-channel match-up and other pretreatments in system operations, have so far obstructed the implementation of a functional laser-microcomb system.

**Fig. 1:**
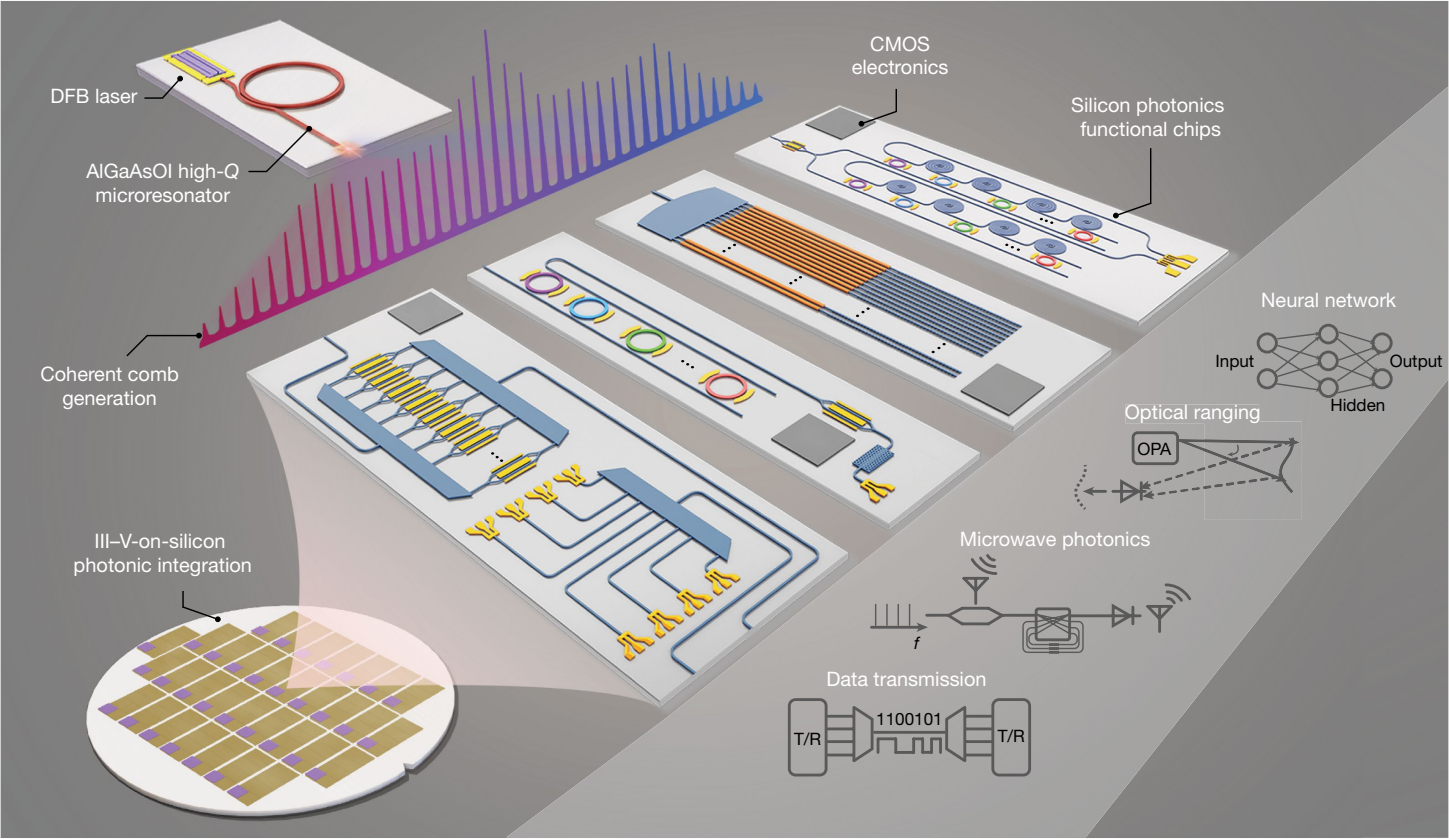
Microcomb-based SiPh optoelectronic systems. Conceptual drawings for several integrated optoelectronic systems (data transmission, microwave photonic signal processing, optical beam steering and photonic computing) realized by combining a microcomb source with silicon photonic chips. With III–V-on-silicon photonic integration, the chips are expected to contain all the essential functions (for example, laser-microcomb generation, passive and active optical components, and the electronics for supporting signal processing and system control).

Here we make a key step in combining these two essential technologies. Using an aluminium gallium arsenide (AlGaAs)-on-insulator (AlGaAsOI) microresonator that can be directly pumped by a DFB on-chip laser, a dark-pulse microcomb is generated, which exhibits state-of-the-art efficiency, simple operation and long-time stability. Such a coherent comb is used to drive CMOS-foundry-based SiPh engines containing versatile functionality, which can be used for a wide range of applications (Fig. 1). On the basis of this approach, system-level demonstrations are presented for two major integrated photonics fields. (1) As a communications demonstration, we present a microcomb-SiPh transceiver-based data link with 100-Gbps pulse-amplitude four-level modulation (PAM4) transmission and 2-Tbps aggregate rate for data centres. (2) For microwave photonics, a compact microwave filter is demonstrated with tens-of-microseconds-level reconfiguration speed by an on-chip multitap delay-line processing scheme, whose tunable bandwidth and flexible centre frequency are capable of supporting fifth-generation (5G), radar and on-chip signal processing. This work paves the way towards the full integration of a wide range of optical systems, and will significantly accelerate the proliferation of microcombs and SiPh technologies for the next generation of integrated photonics.

## Building blocks

### AlGaAsOI microcombs

**Fig. 2:**
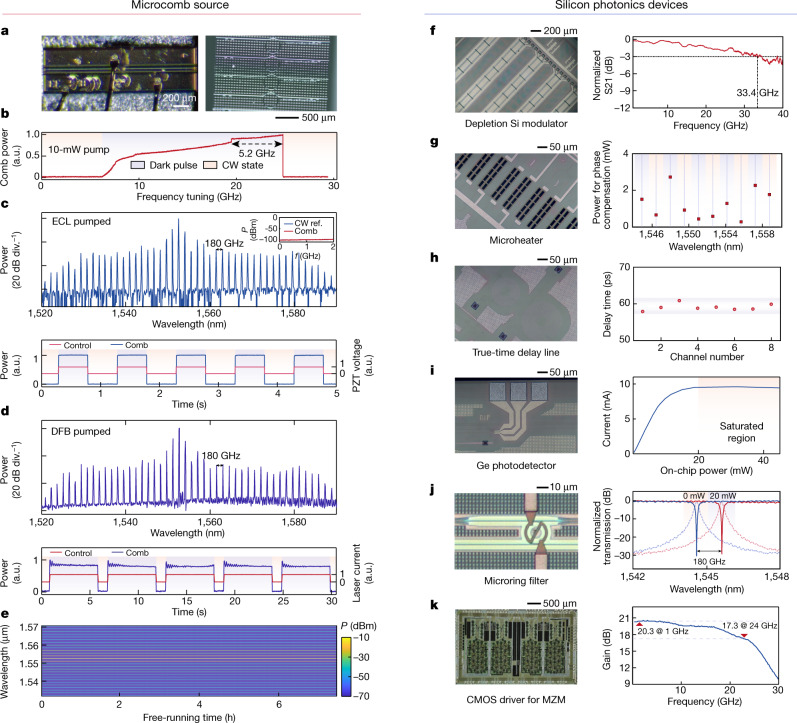
Comb generation and fundamental characteristics. **a**, Optical image of the InP DFB laser chip and the AlGaAsOI microresonators for dark-pulse generation. **b**, Normalized comb power when tuning the pump frequency across the resonance at around 1,552 nm. With 10-mW pump power, a dark-pulse Kerr comb could be accessed in a large frequency window (tens of gigahertz). CW, continuous wave. **c**, **d**, Two-FSR dark-pulse spectra (top) and the ‘turnkey’ behaviours (bottom) pumped by a commercial external laser (**c**) or a DFB laser chip (**d**) with an equal on chip power of 10 mW. A pair of flat wings besides the pump is formed in both spectra, exhibiting typical profile of the coherent dark-pulse microcombs. Inset: comb intensity noise (resolution BW of 100 kHz). The intensity noise of the dark-pulse Kerr comb is at the same power level as the electrical spectrum analyser background. P, power; f, frequency; PZT, lead zirconate titanate. **e**, Long-term stability of a free-running comb. **f**–**k**, Optical images and main performance of several Si-based fundamental devices, including a depletion-mode Si MZM (**f**), a TiN microheater (**g**), a Si spiral waveguide delay line (**h**), a vertical epitaxial Ge PD (**i**), a microring filter (**j**) and a CMOS driver for MZMs (**k**). More details can be found in Methods.

The integrated comb source used in this work is based on an AlGaAsOI platform^37^ by heterogeneous integration, as shown in Fig. 2a. Combined with the extremely high third-order nonlinear coefficient of AlGaAs (*n*_2_ ≈ 2.6 × 10^−17^ m^2^ W^−1^), Kerr comb generation from the AlGaAsOI microresonators (Fig. 2a, right) with a moderate quality (*Q*) factor (one million to two million) exhibits a record-low parametric oscillation threshold down to tens of microwatts and coherent comb-state generation under pump power at the few-milliwatts level, which can be satisfied by a commercial indium phosphide (InP) DFB laser chip (Fig. 2a, left).

Besides efficiency, the operation simplicity and stability of the comb source are also critical for practical applications. In the anomalous dispersion regime, a special type of bright soliton, termed ‘soliton crystals’^38^, exhibit these features to support system-level demonstrations without relying on electronic control^14,16,39^. In this paper, a dark-pulse state^40,41^ is used to achieve coherent microcombs. This state works in the normal dispersion regime with the assistance of the avoided mode-crossing effect (Supplementary Note I). The dark-pulse operation experiences a much smaller power step during the transition to the coherent comb state (Supplementary Note II). More importantly, owing to the thermo-induced self-stable equilibrium mechanism of microcavities, the strong thermo-optic effects of AlGaAs (2.3 × 10^−4^ K^−1^) here can be leveraged to significantly extend the accessibility window of the coherent comb state^42^. Such behaviour is experimentally characterized in Fig. 2b, where the comb power versus pump detuning is recorded, showing the accessible frequency range of the dark pulse to tens of gigahertz, about ten times wider than that with bright solitons^33^.

Together, these traits make coherent comb generation efficient and robust in AlGaAsOI microresonators, with greatly simplified operation. Figure 2c, d shows the dark-pulse spectra pumped by an external cavity laser and a DFB laser chip, respectively, with the same on-chip power of 10 mW. Such a state can be deterministically triggered by simply turning on the laser without relying on any tuning control of electronics, thus showing ‘turnkey’ behaviour (Methods). Moreover, benefiting from the self-stabilization enabled by the strong thermo-optic effect, the comb is able to maintain stable operation without feedback loops. Figure 2d shows the spectral power versus time in a free-running AlGaAs dark pulse, with small power fluctuations over 7 h. The simplicity of both generation and stabilization facilitates seamless implementation of AlGaAsOI microcombs in current optoelectronic systems and are well suited for practical applications.

### Silicon photonic engines

A monolithic SiPh circuit is used to process the generated comb lines for diverse optoelectronics systems. Such ‘silicon photonic engines’ provide functionality such as filtering, modulation, multiplexing, time delay and detection on the same chip. Figure 2fk shows the essential photonic building blocks of the optical processing engines and their key performance metrics. For signal encoding, Mach–Zehnder interferometer (MZI) travelling-wave PN depletion modulators with >33-GHz electro-optical bandwidth are used (Fig. 2f). Heaters are used to match up the modulators with the comb channels by thermal tuning (Fig. 2g). A representative result for such phase compensation in a modulator at different channel wavelengths is shown in Fig. 2g (left). To implement on-chip true-time delays, spiral waveguides with adiabatic bends are designed, as shown in Fig. 2h. The deviation of 60-ps delay lines is within 3 ps. Figure 2i shows the germanium (Ge) photodetector (PD) with about 0.5–0.8 A W^−1^ at different on-chip power levels, and with a saturation power of approximately 20 mW. A microring filter array is used here to control the comb lines individually, as shown in Fig. 2j. A 180-GHz-wide (2 free spectral range (FSR)) channel-selecting range can be obtained with 20-mW heater power (Methods). In addition, the SiPh devices support system-level assembly with electronic integrated chips (Fig. 2k), allowing future integration of low-noise trans-impedance amplifiers and high-speed drivers.

### System demonstrations

Next, two pivotal system-level demonstrations are presented: (1) a microcomb-based integrated photonic data link with a greatly increased data rate compared with traditional Si-based transceivers and (2) a rapidly reconfigurable microcomb-based microwave photonics filter with a high level of integration.

### Parallel optical data link

**Fig. 3:**
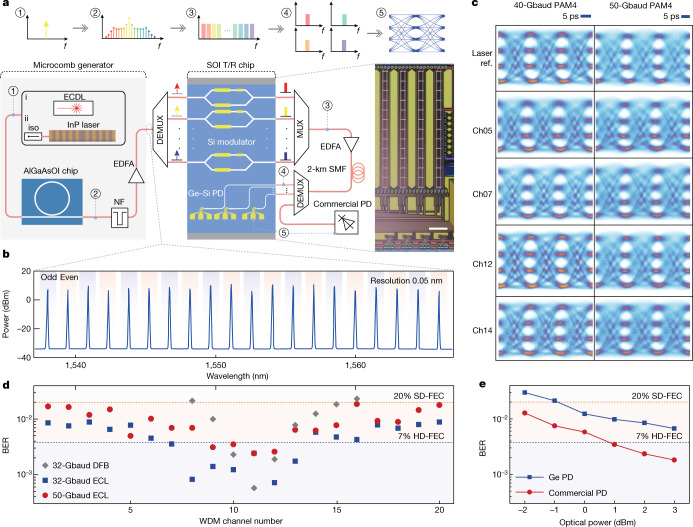
Transmission results. **a**, Schematic of the microcomb-based data transmission set-up. The dark-pulse Kerr comb source is pumped by a continuous-wave laser, which can be generated by a commercial external cavity diode laser (ECDL, i) or a distributed feedback laser chip (ii). The generated comb is then sent into a SiPh T/R chip. iso, isolator; NF, notch filter; DEMUX, demultiplexer; MUX, multiplexer. Scale bar, 500 μm. **b**, A 20-line comb spectrum in the C band as the multiwavelength source before injection into the SiPh T/R chip. **c**, Typical eye diagrams of the chosen channel after modulation by SiPh modulators at different symbol rates (32 Gbaud, 40 Gbaud and 50 Gbaud). **d**, BER for each comb line. The blue squares and red circles indicate the ECL-pumped comb data transmission results at symbol rates of 32 Gbaud and 50 Gbaud, respectively. All channels are considered within the given HD-FEC (3.8 × 10^−3^) or SD-FEC (2 × 10^−2^) threshold (blue and orange dashed lines, respectively). The grey diamond markers show the performance when pumping the AlGaAs microresonator with a DFB chip. The wavelength-dependent BERs mainly result from the increased noise of the pre-amplifier at the edge of its operation band. The optimized receiving power for each channel is about 2–3 dBm. **e**, BER versus receiving power comparison between an on-chip Ge–Si PD and a commercial PD with the variation of the receiving power. The main limitation of the Ge–Si PD is the non-optimized frequency response (Methods).

A schematic of the PAM4 WDM transmission system is shown in Fig. 3a. The channel spacing of the AlGaAsOI dark-pulse comb can be reconfigured from 1-FSR to multi-FSR via appropriate pre-calibration of the start-up setup (for example, laser detuning, temperature and so on)^41^. To achieve a higher average optical carrier-to-noise ratio while providing sufficient channel counts within the operation band, a 2-FSR spacing comb is selected as the WDM source here owing to its higher comb-line power. For pumps, a DFB laser chip and a commercially available external cavity laser (ECL) pump source are used, respectively. After the comb generation, an amplifier is needed to compensate the extra penalty brought by the demultiplexing and coupling loss. The spectrum, after amplification, is shown in Fig. 3b, in which 20 consecutive comb modes (from 1,537 nm to 1,567 nm, about 3.75-THz wide) are displayed with <5-dB power difference with proper thermal pre-setting (Methods). A simplified scheme is used to verify the chip-scale data transmission capability for carrying multi-terabits per second. The comb lines are filtered out and split into odd and even test bands by a wavelength selective switch (WSS) and then launched into the SiPh transmitting-recieving (T/R) chip, including Si modulators and Ge photodiodes. On each WDM channel, the SiPh modulators encode the carrier into PAM4 signal format at symbol rates from 32 Gbaud to 50 Gbaud. Figure 3c shows representative examples of eye diagrams after traversing 2-km-fibre links. At the receiving side, the signal is partly coupled to an on-chip Ge photodiode, whereas the remaining part is sent into a commercial PD for performance comparison. The bit-error ratio (BER) of each channel is calculated after direct detection (Methods).

Such a dense wavelength division multiplexing scheme can greatly improve the aggregate bit rate while maintaining excellent scalability. In our proof-of-concept demonstrations, 20 comb lines in the C-band are used as the source. Figure 3d shows the BER results under three scenarios: (1) 32-Gbaud and (2) 50-Gbaud PAM4 with an ECL pump, and (3) 32-Gbaud PAM4 with a DFB pump. Considering the ECL-pumped microcomb, 7(4) channels are below the 7% hard-decision forward error correction (HD-FEC) threshold at the symbol rate of 32(50) Gbaud, with the remaining channels below the 20% soft-decision forward error correction (SD-FEC) threshold. In this case, the microcomb-based SiPh transmitter enables a baud rate of 50 Gbaud per single lane, corresponding to an aggregate bit rate of 2 Tbit s^−1^ (1.65 Tbit s^−1^ net rate after FEC overhead subtraction). For a higher-level integrated system, the commercial ECL pump is replaced by a DFB laser chip. With the integrated pump source, the transmitter achieves a total data transmission rate of 448 Gbit s^−1^, with 7 channels under the FEC threshold. Another advantage of SiPh is the possibility of integrating the transmitter and receiver. BER results after optical to electrical (O/E) conversion by both commercial III–V photodiodes and on-chip Ge photodiodes are shown in Fig. 3e. At the 20% SD-FEC threshold, the penalty between two devices is approximately 2.3 dB at 32 Gbaud (Methods).

### Reconfigurable microwave photonic filter

**Fig. 4:**
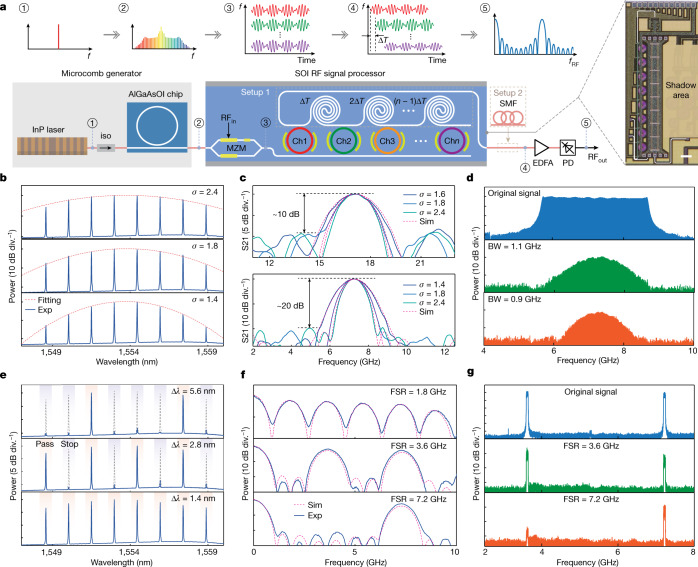
Reconfigurable MPF results. **a**, Schematic of the setup to perform microcomb-based reconfigurable MPF. The time delays between comb lines are produced by on-chip spiral delay lines (setup 1) and dispersive propagation from a spool of SMF (setup 2). Scale bar, 200 μm. **b**, Optical spectra of Gaussian-apodization comb lines for BW programming (*σ*, Gaussian factor; blue, experiment (Exp); red, Gaussian fitting). **c**, RF filtering responses of the MPF with various passband BWs, based on the setup 1 (top) and setup 2 (bottom). The red dashed curves show the theoretical fitting results (Sim.) (Supplementary Note III). **d**, Proof-of-concept demonstration of RF filtering of a wideband RF signal. From top to bottom: RF spectra of original signal, signal after 1.1-GHz BW filter and signal after 0.9 GHz BW filter. **e**, **f**, Optical spectra (**e**) and corresponding RF responses (**f**) of the MPF with various FSRs, produced by modifying the comb line spacing and based on setup 2. *∆λ*, wavelength distance between adjacent comb lines. **g**, Proof-of-concept demonstration on RF filtering of a complex dual-channel RF signal. From top to bottom: RF spectra of original signal, signal after 3.6-GHz FSR filter and signal after 7.2-GHz FSR filter.

The reconfigurable microwave photonic filter (MPF) is constructed using a tapped delay line (TDL)^43^. It is worth mentioning that TDL-based MPFs can follow two approaches depending on whether the tap delays are produced by non-dispersive (true-time) delay lines^44^ or dispersive delay lines^13,45–47^. In this work, both approaches are implemented. A schematic of the experimental setup is shown in Fig. 4a. The 180-GHz-spacing microcomb served as taps for the MPF. The comb lines are then manipulated by a SiPh signal processor containing a high-speed Mach–Zehnder modulator (MZM), an eight-channel add-drop microring array (MRA) and spiral delay lines. The input radio frequency (RF) signal is loaded by the MZM. The MRA here acts as an on-chip optical spectral shaper (OSS) for the comb lines, performing spectrum slicing, line-by-line pulse shaping (weighting on taps) and spectrum recombination in sequence. A cluster of spiral waveguides offers a fixed time delay (Δ*T*) between adjacent taps. Finally, the processed comb lines are beaten in an off-chip fast PD to synthesize the RF filtering profiles.

The system shows flexible reconstruction features in terms of passband bandwidth (BW) and RF FSR. Figure 4b depicts the optical spectra using Gaussian apodization on comb lines for passband BW reconfigurability^46^. The corresponding RF filtering responses are given in Fig. 4c, with non-dispersive delay (top) and dispersive delay (bottom) configurations, respectively. The 3-dB BW of the MPF in the non-dispersive delay scheme can be continuously adjusted within a range of about 1.97–2.42 GHz by tuning the Gaussian parameter *σ* from 2.4 to 1.6. The main-to-sidelobe suppression ratio is about 10 dB. Better performance (>20-dB main-to-sidelobe suppression ratio) is achieved using the dispersive delay scheme, with a subgigahertz-level filtering BW tunability. The results in Fig. 4e, f show the reconfigurability of RF FSR by modifying the comb line spacing: comb line spacings of 5.6 nm, 2.8 nm and 1.4 nm result in RF filtering response FSRs of 1.8 GHz, 3.6 GHz and 7.2 GHz, respectively. In contrast with other state-of-the-art microcomb-based MPFs using either bulk OSS^46,47^ or changing soliton states^13^, this work significantly advances the degree of integration and the reconfiguration speed (about 53 μs; Methods), which are crucial for modern wireless communications and avionic applications.

As a paradigm demonstration towards real-world applications, RF filtering on a practical microwave signal is illustrated in Fig. 4d, g. First, a broadband RF signal covering from 5.5 GHz to 9 GHz is shaped by changing the MPF BW from 0.9 GHz to 1.1 GHz, as shown in Fig. 4d, exhibiting reconfigurable passband widths. Moreover, to validate the FSR reconfigurability, a RF test signal is generated with a 50 Mb s^–1^ quadrature phase shift keying (QPSK) modulation at 3.6 GHz and 7.2 GHz, respectively (Fig. 4g). It can be observed that by setting the proper tap spacing with the on-chip OSS, the signal at 3.6 GHz could be optionally rejected.

## Discussion

The performance of these systems can be further improved by optimizing the integrated devices or employing superior signal-processing techniques. Additional multiplexing techniques (such as space-division multiplexing and polarization-division multiplexing) and higher modulation formats (such as, PAM6 and PAM8) could be used to boost the transmission capacity. The data rate can be further scaled up to >10 Tbps by broadening the operation wavelength to the L band and the S band. The performance of the DFB-pumped integrated comb source is mainly limited by the relatively high noise floor of the free-running DFB laser (Methods), which lowers the optical signal-to-noise ratio (OSNR). For the RF filter, a narrower filtering BW (down to subgigahertz) and a higher tuning resolution can be obtained by increasing the number of tap channels used in the finite impulse response configurations^43^, that is, expansion of the MRA.

We expect more integrated functionality be incorporated in the future, culminating in fully integrated microcomb-based optoelectronic systems. For instance, self-injection locked dark-pulse microcomb sources^21^ could be monolithically realized by using heterogeneously integrated III–V lasers and microresonators^23^. The discrete erbium-doped fibre amplifiers (EDFAs) could be replaced by on-chip SOAs, which can potentially be integrated with other photonic components on the same chip^48,49^. More recently, AlGaAs-on-SOI photonic circuits have been developed to integrate the two material platforms we used in this work on the same wafer^50^. The photonic elements can also be combined with application-specific electronic circuits, which will further improve the compactness and power efficiency. Considering the versatility offered by the technologies, microcomb-driven SiPh systems will provide a mass-produced and low-cost solution to a broad range of optoelectronics applications, therefore facilitating the next generation of integrated photonics.

## Methods

### Design and fabrication of the devices

The ring waveguides of the AlGaAsOI resonators were designed to work within the normal dispersion regime in the C band, with dimensions of 400 nm × 1,000 nm. The width of the bus waveguide at the facet was designed to be 200 nm for efficient chip-to-fibre coupling. The fabrication of the AlGaAs microresonators was based on heterogeneous wafer bonding technology. The process is currently realized at the 100-mm-wafer scale without any strict fabrication processes such as chemical-mechanical polishing or high-temperature annealing that are not compatible with the CMOS process. It can therefore be directly adopted by current III–V/Si photonic foundries^51^. A *Q* factor >2 million can be obtained in the AlGaAsOI resonator, corresponding to a waveguide loss of <0.3 dB cm^−1^. The fraction of aluminium is 0.2, which corresponds to a two-photon absorption wavelength of around 1,480 nm. The epitaxial wafer growth was accomplished using molecular-beam epitaxy. A 248-nm deep-ultraviolet stepper was used for the lithography. A photoresist reflow process and an optimized dry etch process were applied in waveguide patterning to minimize waveguide scattering loss. More fabrication details can be found in refs. ^52^^,^^53^. The SiPh PIC, including its Si modulators and Si–Ge PDs, was fabricated on a 200-mm SOI wafer with a Si-layer thickness of 220 nm and a buried oxide layer thickness of 2 μm using CMOS-compatible processes at CompoundTek Pte in a one-to-one 200-mm-wafer run with its standard 90-nm lithography SOI process. The waveguide loss in this SiPh platform is approximately 1.2 dB cm^−1^ in the C band. In our experiment, lensed fibres with different mode field diameters were selected for the AlGaAsOI and SOI chips; the coupling loss is about 3–5 dB per facet for AlGaAsOI waveguides and about 2–3 dB per facet for Si waveguides.

### Characterizations of the building-block units

The FSR of the 144-μm-radius rings utilized in this study is about 90 GHz. The microcomb shows advances both in start-up and stabilization. During the dark-pulse generation, a much smaller abrupt power change occurs when the comb transits from continuous-wave states to dark-pulse states, indicating the elimination of the well known triggering problem in bright soliton generation. Compared with general bright solitons, the dark pulse is inherently tolerant to thermal effects that usually make bright soliton states difficult to access^54^. For long-term stability measurement, the comb spectra and comb line power of a free-running dark-pulse comb are recorded by a high-resolution optical spectrum analyser (OSA) every 5 min.

More details are presented here for the SiPh devices shown in Fig. 2. The opto-electrical BW of the depletion-mode Si-based MZMs was measured by a vector network analyser (Keysight N524), with the typical results of >30 GHz. The on-chip phase compensation units are MZI-based titanium nitride (TiN) microheaters. The resistance is approximately 200 Ω. The TiN metal layer is about 1 μm above the Si layer, ensuring a heating efficiency of about 20 mW π^−1^. Meanwhile, a deep trench process is utilized to isolate each microheater to diminish thermal cross-talk. For the on-chip true-time delay line, we adopted a 2-μm-wide multimode Si waveguide for low-loss transmission. Euler curves were used in the spiral waveguide for adiabatic bending. For a 60-ps Si delay line, the total loss is <0.5 dB, with a delay-time variation of <3% among 8 tested devices. For the vertical epitaxial Ge PD, the responsivity declines with the increasing on-chip power. A saturated point of about 20 mW could be reached when the power is further increased. Microring filters employed for WDM could be tuned by microheaters, with which a 180-GHz channel spacing can be obtained under 20-mW power dissipation. The CMOS drivers for signal amplification before injection into the Si MZM (not used in the high-bit-rate (>50 Gbps) signal transmission experiment) show a 3-dB gain BW of about 24 GHz.

**Extended Data Fig. 1:**
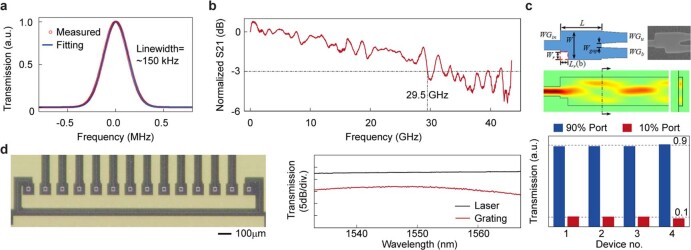
Performance of building block devices. **a**, Measured linewidth of the DFB laser. **b**, The measured 3dB bandwidth of the Si-Ge PD photodiode. **c**, Design, fabrication and measurement results of the 10:90 asymmetric MMI as on-chip monitor for silicon photonics engines. **d**, Optical image of the grating coupler (left) and its normalized transmission.

The performance of other building-block devices is presented in Extended Data Fig. 1. The linewidth of the DFB laser used as the pump is measured by a delayed self-heterodyne method^55^. The measurement and Lorentzian fitting result are shown in Extended Data Fig. 1a, exhibiting a laser linewidth of about 150 kHz. For the SiPh devices, the 3-dB BW of the Si–Ge photodiodes is shown in Extended Data Fig. 1b, indicating an approximately 30-GHz S21 parameter. Such a non-optimized BW accounts for the penalty in Fig. 3c. Structure design for a lower resistor-capacitor time constant could further increase the operation BW. For on-chip monitoring, the asymmetric MMI-based 10:90 power splitter^56^ is employed in the system, as shown in Extended Data Fig. 1c. The symmetry of the multimode region is broken by removing the corner of the MMI (marked with a red dashed rectangle), which causes a dramatic redistribution of the optical field, thus leading to an uneven power splitting by changing the width of the cut-off corner. We randomly chose four identical 1:9 MMIs and tested the power splitting ratios. The results were found to be close to the design target (dashed line), exhibiting good consistency, as shown in the bottom panel of Extended Data Fig. 1c. Moreover, the grating couplers used in this work (Extended Data Fig. 1d) show a roughly 2-dB coupling efficiency difference across the operation band (1,535–1,565 nm).

### Turnkey dark-pulse microcomb generation

**Extended Data Fig. 2:**
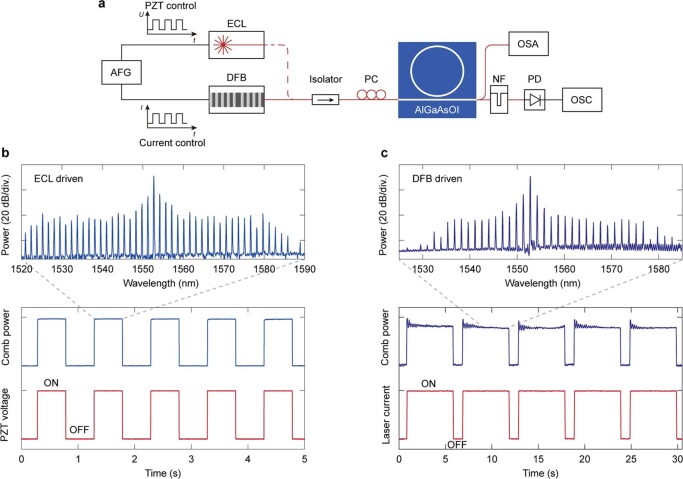
Turnkey AlGaAs dark-pulse microcomb generation. **a**, Experimental setup. **b**, ECL and **c**, DFB laser chip driven comb spectra and the comb power variations along with the control signal in five consecutive switching tests.

The turnkey microcomb generation test setup is shown in Extended Data Fig. 2a, with either an ECL or a DFB laser as the pump. Slow laser-frequency detuning is enough for microcomb generation, which can be realized by adjusting the cavity length via tuning the lead zirconate titanate voltage of the commercial ECL or changing the laser current of DFB, respectively. After the comb generation, the spectra are recorded; meanwhile, the total power of the generated comb lines is measured in real time. A pre-calibration process is required to ensure the laser frequency will locate at the comb accessing range ultimately. For the ECL-pumped dark-pulse comb (Extended Data Fig. 2b), a 1-Hz square wave is used as the trigger signal, which tunes the pump wavelength about 0.3 nm away from or into the resonance. For the DFB-pumped experiments (Extended Data Fig. 2c), when a laser is turned on, there is always an automatic frequency ramping-up process owing to the injected carrier and the warming of the cavity, which can directly initiate the microcomb generation as long as the lasing frequency of the final stable state lies within the range of the access window of the coherent state. In our experiment, the laser current is switched between two values with a period of 6 s (1 s for the ‘off’ state and 5 s for the ‘on’ state). Both results show immediate on–off behaviours of microcomb generation along with the low-speed control signal. It is noted that there is some power ripple of the DFB-pumped comb in the first few seconds, which is due to the temperature vibration caused by thermoelectric cooler, after which the comb state is stabilized. The comb is reproducible in several consecutive switching tests, with great robustness.

### Details of data transmission experiments

**Extended Data Fig. 3:**
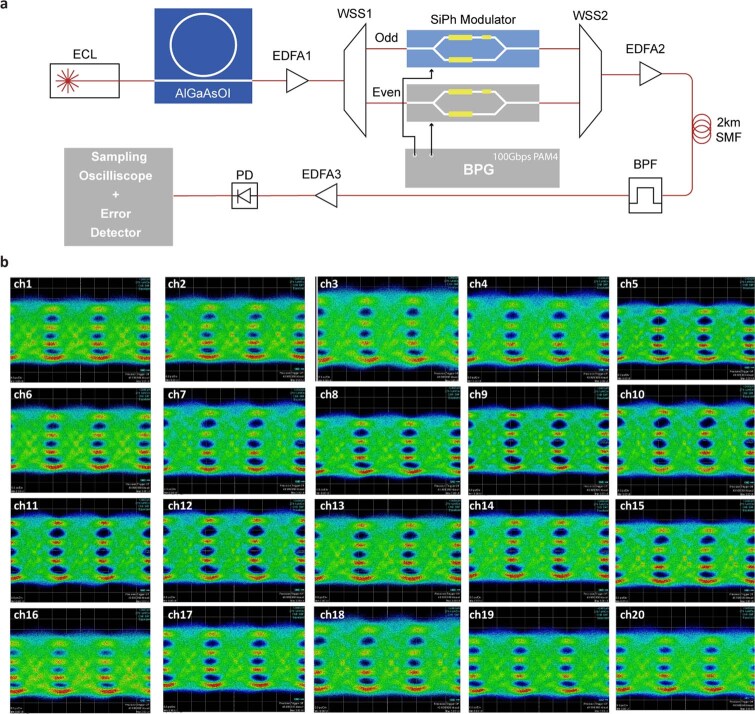
Detailed information for data transmission. **a**, Detailed experimental set-up for the odd/even test band for the comb-based silicon photonics data link. **b**, measured 100 Gbps PAM-4 eye diagrams from the sampling oscilloscope for each channel at receiving end.

In our experiment, the microcomb is first pumped by a commercial tunable laser (Toptica CTL 1550), then by a DFB laser chip for a higher degree of integration, where an optical isolator is deployed between the DFB laser and the AlGaAsOI microresonator to eliminate the reflection. When tuning the pumping wavelength from the blue side to a certain detuned value at around 1,552.5 nm, both configurations generate dark pulses with 2-FSR comb spacing. The detailed experimental setup for data transmission is shown in Extended Data Fig. 3a. For the comb spectrum with large power fluctuations, an additional amplification process is required owing to the insufficient gain of those low-power channels, which introduces extra system complexity and power consumption on the transmitting side. In this work, owing to the strong thermal effect, the avoided mode-crossing (AMX) strength of the AlGaAs microresonator can be thermally pre-set to obtain a coherent microcomb with a less disparate power distribution across the operation band. Thus, only a notch filter is required to attenuate the central three comb lines for the subsequent equalized comb amplification. The comb is amplified by an EDFA and then split into odd and even test bands^39,57,58^ by a wavelength-selective switch (Finisar Waveshaper 4000s). A Si modulator and a lithium niobate (LN) modulator (EOspace, 35-GHz BW) are deployed at the odd and even bands, respectively. Ten comb lines in each test band are simultaneously modulated. The modulators are driven at a 32-Gbaud or 50-Gbaud symbol rate. The differential PAM-4 signal is generated by a commercial pulse pattern generator (Anritsu PAM4 PPG MU196020A). The insertion loss of the SiPh (LN) modulator is 13(8) dB. The SiPh modulator undergoes a relatively high loss (including the edge coupling loss of about 2 dB per facet), which results in a power difference between the two test bands. The modulated test bands are then combined by a 50:50 power coupler and launched into another WSS for comb power equalization. At the receiving side, each WDM channel encoded by the Si modulator is sequentially filtered out and measured. Eye diagrams are produced by a sampling oscilloscope (Anritsu MP 2110A) with a 13-tap transmitter and dispersion eye closure quaternary (TDECQ) equalizer (accumulation time, 8 s). The BERs are measured online by an error detector (Anritsu PAM4 ED MU196040B) with 1-dB low-frequency equalization and a decision-feedback equalization. Extended Data Fig. 3b shows the 100-Gbps PAM4 eye diagrams for each of the 20 channels.

It is worth noting that the performance is underestimated. In our proof-of-concept test configuration, ten channels in each test band are modulated at the same time. Considering two-photon absorption in Si waveguides, the maximum input power for the Si modulator is about 13 dBm, which results in only 3-dBm optical power per single lane. Moreover, considering the extra penalty introduced by the WSS for power equalization, unnecessary in real-word transmission scenarios, the OSNR for each channel can be at least 10 dB higher. Thus, a better transmission result is attainable.

### Noise analysis of different pump schemes

**Extended Data Fig. 4:**
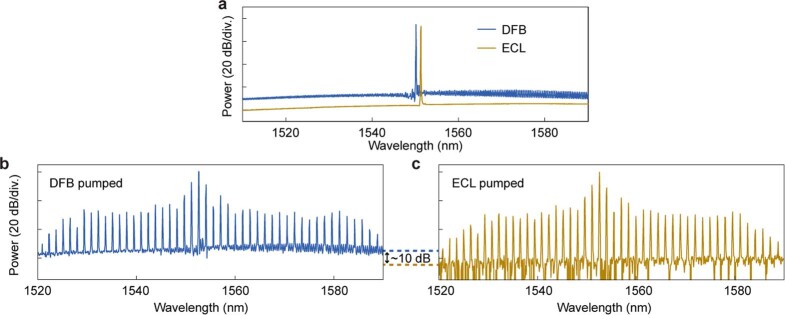
Microcomb generation with different pump schemes. **a**, Measured linewidth of the DFB laser. Comparison of the comb spectra pumped by **b**, a commercial external cavity laser and **c**, a DFB laser chip.

The noise floor of the DFB and the ECL are roughly characterized in an OSA, as shown in Extended Data Fig. 4a. The laser spectra indicate that the noise of the DFB is evidently higher than that of the ECL. The combs in our experiments are pumped by the free-running DFB laser and the ECL separately, as shown in Extended Data Fig. 4b, c. With the almost same pumping power of about 10 mW, the DFB chip holds a 10-dB-higher noise floor compared with the ECL, corresponding to an equivalent OSNR reduction in each comb line. Moreover, the amplification after the comb generation would also result in OSNR degradation, which could be a potential problem when replacing the current EDFA with integrated SOAs (about 4–5-dB-noise-floor increment in a commercial EDFA and about 7 dB in commercial on-chip SOAs).The OSNR of the DFB-pumped microcomb can be further improved by employing an on-chip optical filter for comb distillation^59,60^ or introducing optical injection locking between the microcomb and slave lasers for low-noise amplification^61^. Also, increasing the pump power will lead to a higher average OSNR and more stable long-term behaviour, which is an advantage over the injection-locking-based dark-pulse generation^21,62^.

### Setup of the dispersive delay-line MPF scheme

**Extended Data Fig. 5:**
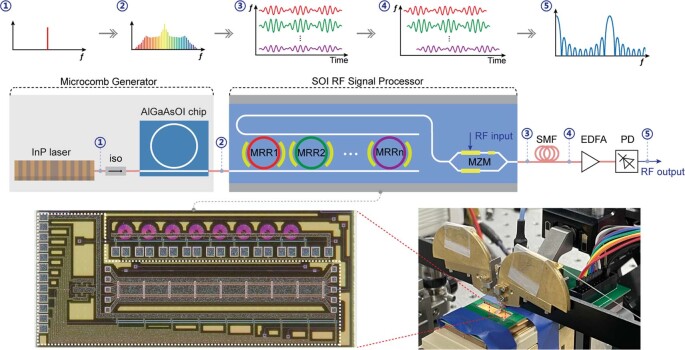
Dispersive delay-line MPF. Experimental setup of the second dispersive delay-line scheme based TDL-MPF.

As the non-uniformity of delays owing to the inevitable fabrication errors will degrade the filtering performance, the second TDL-MPF approach is also implemented to further determine the optimal filtering performance: a spool of single-mode fibre (SMF) is used instead of the on-chip spiral delay lines to produce dispersive delay. Extended Data Fig. 5 shows the experimental setup of the reconfigurable MPF carried out in a dispersive delay-line configuration. Compared with Fig. 4a, most of the MPF system remains unchanged and has one main difference, which is that the on-chip true-time spiral delay lines are removed from the SiPh signal processor. The processed comb lines will propagate through a spool of 5-km SMF (as a dispersive element) to obtain a solid delay unit between adjacent taps, which can be expressed as *T* *=* δ*λDL* (ignoring the high-order dispersion of SMF), where δ*λ* represents the comb line spacing, *D* is the dispersion coefficient of SMF and the *L* is the length of SMF. In this scheme, the basic delay *T* among comb lines is generated by a single dispersive element, which can be kept as uniform value and not influenced by fabrication errors. Besides, this system is more flexible; for instance, the centre frequency of the filtering passband can be adjusted by simply change the length or dispersion coefficient of SMF.

### Details of RF filter experiments

The DFB-driven dark-pulse Kerr comb exhibits 2-FSR (180-GHz) comb spacing. The initial comb source is amplified by an EDFA, and 8 comb lines in the range of 1,547–1,560 nm are selected using an optical bandpass filter before injection into a SiPh signal processor chip. The input and output coupling are achieved via grating couplers of about 40% coupling efficiency. Frequency-swept RF signals with 9-dBm power from a vector network analyzer are applied to the Si MZM in double-sideband format. The tap weighting coefficients are set by adjusting the relative detuning among the comb lines and their corresponding resonance wavelengths in the Si MRA with TiN microheaters placed on the waveguides. The output light of the Si chip is split by a 10:90 optical power coupler: 10% of the light is sent into an optical spectrum analyser (Yokogawa AQ6370C) for spectral monitoring, whereas the other 90% of the light propagates through the follow-up optical link. In the dispersive delay scheme, a spool of 5-km SMF is used to acquire the dispersive delay between adjacent comb lines (taps). Finally, the processed comb lines are beat in a 50-GHz PD (Finisar 2150R) to convert the optical signal into electrical domain. A low-noise EDFA is placed before the PD to compensate for the link insertion loss and coupling loss.

For the practical demonstrations of RF signal filtering, a 50 Gsamples s^−1^ arbitrary waveform generator (AWG, Tektronix AWG70001) is used to produce the desired RF input signals. To validate the BW reconfigurability of this filter, an ultrawideband RF signal is generated, spanning from 5.5 GHz to 9 GHz. To validate the FSR reconfigurability of this filter, a complex RF signal is produced that contains a 50-Mb-s^−1^ QPSK spectrum modulated at 3.6 GHz and a 50-Mb-s^−1^ QPSK spectrum modulated at 7.2 GHz. The RF outputs from the AWG are amplified by a linear electrical driver (SHF 807C) before routing to the Si MZM. The filtered RF signals are detected by a signal analyser (Keysight N9010B) for spectrum measurement. A similar FSR multiplication of the MPF has been reported previously and explained by temporal Talbot effects^63^. However, the crucial Talbot processor used in these MPF systems is based on more complex discrete devices, which will increase the power dissipation and make the system less stable.

**Extended Data Fig. 6:**
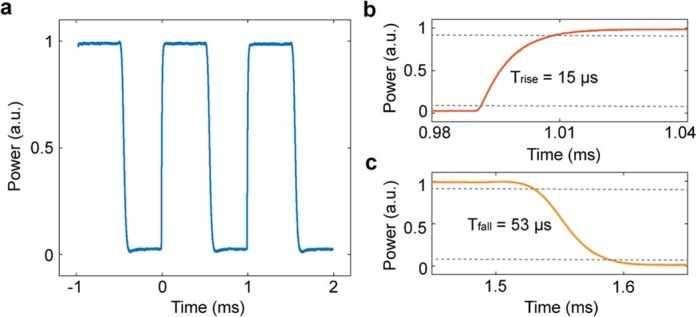
The response time measurement for the reconfiguration of RF filtering profiles. **a**, Measured temporal response of the MRR under a square-wave electrical signal driving. **b**, MRR switched from minimum to maximum transmission, and **c**, maximum to minimum transmission. 90/10 rise/fall times are 15 μs and 53 μs, respectively.

Unlike the conventional waveshaper based on bulky liquid-crystal spatial light modulators^64^, one of the remarkable advantages of the chip-scale add-drop microring resonator (MRR) array used in our work is the rapid reconfiguration of RF filtering responses. The reconfiguration operation on filtering spectra is realized by adjusting the shaping profiles of comb lines, through the TiN microheater placed on the waveguides. To explore the maximum reconfiguration speed, a standard electrical square-wave waveform is generated by a function waveform generator (RIGOL, DG2102) to drive a single MRR channel. The output of the MRR is received by a photodetector (Thorlabs DET08CFC/M), and then recorded by a digital oscilloscope (RIGOL, DS7014 10 GSa s^−1^). Extended Data Fig. 6 shows the measured switching temporal response. As seen in Extended Data Fig. 6b, c, the 90/10 rise and fall times are 15 μs and 53 μs, respectively. Therefore, the fastest response speed for the reconfiguration operation is approximately 19 kHz.

## Online content

Any methods, additional references, Nature Research reporting summaries, source data, extended data, supplementary information, acknowledgements, peer review information; details of author contributions and competing interests; and statements of data and code availability are available at 10.1038/s41586-022-04579-3.

## Supplementary information

Supplementary Information This Supplementary Information file contains Supplementary Sections 1–3, including Supplementary Figs. 1–3 and additional references. Section 1: Analysis of the dark pulse evolution. Section 2: Accessibility analysis under the thermal effects. Section 3: Theoretical fitting method for the RF filter responses of the MPF.

## Data Availability

The data that supports the plots within this paper and other findings of this study are available on Zenodo (10.5281/zenodo.6092678). All other data used in this study are available from the corresponding authors upon reasonable request.

## Code availability

The codes that support the findings of this study are available from the corresponding authors upon reasonable request.
