# Supplementary material for: Microcomb-driven silicon photonic systems
Source: Nature. 2022 May 18;605(7910):457–63. doi: 10.1038/s41586-022-04579-3 (PMC9117125; doi:10.1038/s41586-022-04579-3)
Supplement: Supplementary file 1 — This Supplementary Information file contains Supplementary Sections 1–3, including Supplementary Figs. 1–3 and additional references. Section 1: Analysis of the dark pulse evolution. Section 2: Accessibility analysis under the thermal effects. Section 3: Theoretical fitting method for the RF filter responses of the MPF. [file 41586_2022_4579_MOESM1_ESM.pdf]

nature portfolio

https://doi.org/10.1038/s41586-022-04579-3

Supplementary information

Microcomb-driven silicon photonic systems

In the format provided by the authors and unedited

Supplementary Information for

Microcomb-driven silicon photonic systems

Haowen Shu[1,5], Lin Chang[2,5], Yuansheng Tao[1,5], Bitao Shen[1,5], Weiqiang Xie[2], Ming Jin[1] , Andrew Netherton[2], Zihan Tao[1], Xuguang Zhang[1], Ruixuan Chen[1], Bowen Bai[1], Jun Qin[1], Shaohua Yu[1,3], Xingjun Wang[1,3,4,†] and John E. Bowers[2,‡]

[1]State Key Laboratory of Advanced Optical Communications System and Networks, School of Electronics, Peking University, Beijing, 100871, China.

[2]Department of Electrical and Computer Engineering, University of California, Santa Barbara, CA 93106, USA.

[3]Peng Cheng Laboratory, Shenzhen 518055, China.

[4]Frontiers Science Center for Nano-optoelectronics, Peking University, Beijing 100871, China.

[5]These authors contributed equally to this work

Corresponding authors: †xjwang@pku.edu.cn, ‡bowers@ece.ucsb.edu.

Supplementary note I: Analysis of the dark pulse evolution

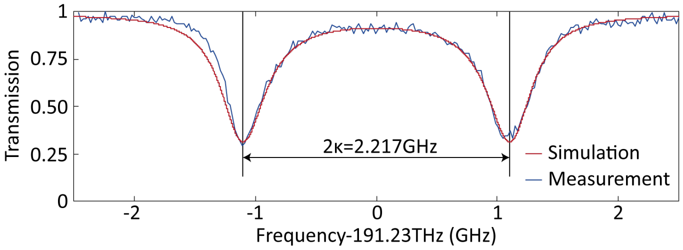

Supplementary Fig. 1: Analysis of the avoid mode crossing. The resonances influenced by avoid mode crossing in experiment and in simulation.

To model the self-stimulation of the dark pulse in an optical microcavity, two mode families have to be considered, where one mode family is the dark-pulse-supporting primary (P) mode family and the other is the auxiliary (A) mode family which exerts the avoided-mode-crossing (AMX) effect on the primary mode family. Two sets of coupled-mode equations are employed here, where the Kerr effects and the linear coupling between two mode families are included [1].

$$t_R \frac{\partial E^{(P)}(t,\tau)}{\partial t} = \left[-\left(\frac{\alpha^{(P)}}{2} - i t_R \delta \right) + iL \frac{\beta_2^{(P)}}{2} \frac{\partial^2}{\partial \tau^2} \right] E^{(P)} + iL\gamma^{(P)} \left| E^{(P)} \right|^2 E^{(P)} + iL\kappa E^{(A)} + \sqrt{\theta} E_{in} \tag{1}$$

$$t_R \frac{\partial E^{(A)}(t,\tau)}{\partial t} = \left[-\left(\frac{\alpha^{(A)}}{2} - i t_R \delta - i\Delta \right) + iL \frac{\beta_2^{(A)}}{2} \frac{\partial^2}{\partial \tau^2} \right] E^{(A)} + iL\gamma^{(A)} \left| E^{(A)} \right|^2 E^{(A)} + iL\kappa E^{(P)} \tag{2}$$

$E^{(P)}$ and $E^{(A)}$ respectively stand for the intracavity temporal fields in the primary and the auxiliary modes, $\alpha^{(P)}$ and $\alpha^{(A)}$ are the roundtrip cavity loss factor, $\beta_2^{(P)}$ and $\beta_2^{(A)}$ represent the second-order dispersion coefficients, and $\delta = \omega_0^{(P)} - \omega_p$ is the detuning, where $\omega_0^{(P)}$ is the resonance frequency of the primary mode and ω_p is the frequency of the pump field. t_R is the roundtrip time of the primary mode and L is roundtrip length. The pump filed is coupled into the primary mode by $\sqrt{\theta} E_{\text{in}}$, where θ is the waveguide coupling coefficient and E_{in} is the pump field. While the coupling between the pump field and the auxiliary mode is ignored, due to the relatively small coupling rate in the pulley couplers. $\gamma^{(P)}$ and $\gamma^{(A)}$

are the nonlinear coefficients. The linear coupling between two mode families is induced by $iL\kappa E^{(x)}(x = A, P)$, where κ is linear coupling strength. Δ indicates the resonant frequency difference between the two modes, which is equal to $t_R \left[\left(\omega_0^{(A)} - \omega_0^{(P)}\right) - i\left(\beta_1^{(A)} - \beta_1^{(P)}\right)\frac{\partial}{\partial\tau}\right]$, where $\omega_0^{(A)}$ is the resonance frequency, β_1^{A} and $\beta_1^{(P)}$ are the first-order dispersion coefficients. $\alpha^{(P)}$, θ, t_R, $\beta_1^{(P)}$ and $\beta_2^{(P)}$ are extracted based on the transmission scan measurement. $\beta_1^{(A)}$, $\beta_2^{(A)}$, $\gamma^{(P)}$ and $\gamma^{(A)}$ are calculated based on the simulation results by an eigeinfrequency solver. κ and $\alpha^{(A)}$ can be estimated relying on the analysis of avoided mode crossing (AMX).

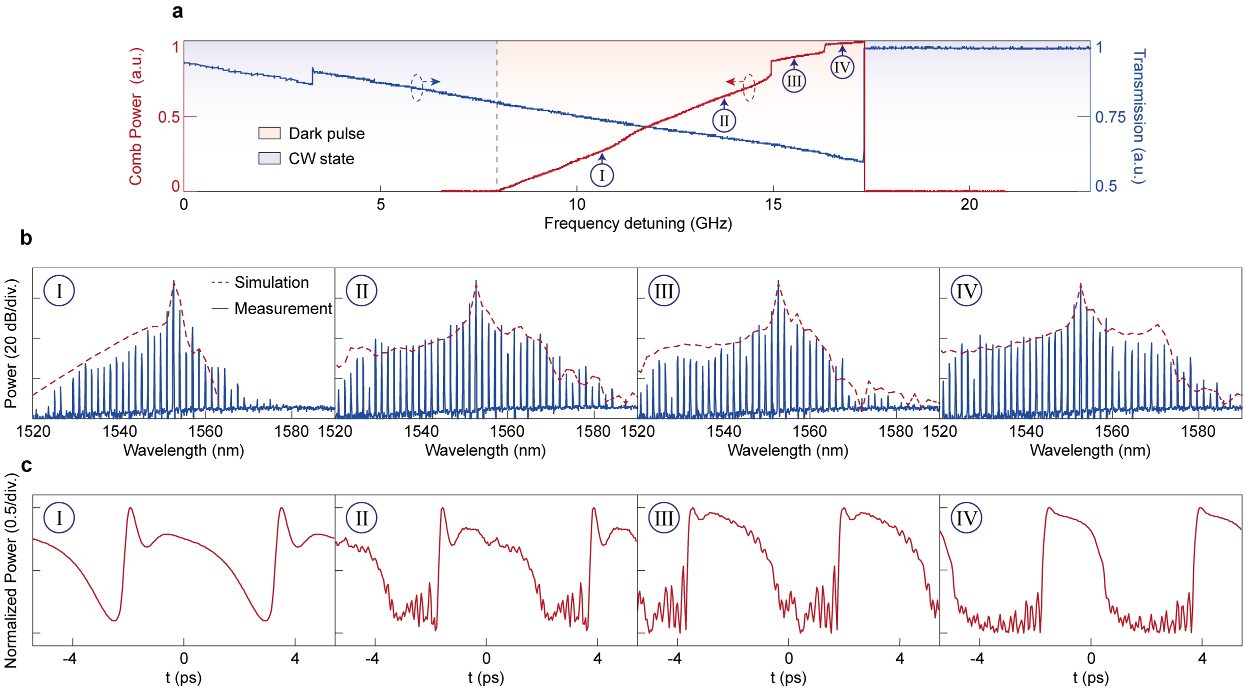

Supplementary Fig. 2: Dark pulse dynamics. **a**, Normalized transmission (blue) and normalized comb power (red) under the pump power of ∼10 mW. The transmission (comb power) is the output power with (without) the pump laser. The continuous wave state and the dark pulse state are marked by blue and red areas respectively. Four spectra are recorded during the evolution of dark pulses, which are marked by I, II, III, and IV. **b**, The spectra of the four points marked in **a**, in experiment (blue) and in simulation (red). **c**, The pulse shapes in simulation of the four points marked in **a**.

Supplementary Fig. 1 shows the two resonances caused by the AMX around 1551 nm. According to the coupling mode theory, the resonance frequencies shifted by the avoided mode crossing can be determined by

$$\omega^{(s,as)} = \left[\omega^{(P)} + \omega^{(A)}\right]/2 \pm \left\{ \frac{\left[\omega^{(P)} - \omega^{(A)}\right]^2}{4} + |K|^2 \right\}^{1/2} \tag{3}$$

Where $\omega^{(P)}$ and $\omega^{(A)}$ are the resonance frequencies without the AMX effect, and $K = L\kappa$ is the coupling rate between the two modes. The AMX strength can be effectively tuned by changing the resonance frequency difference $\omega^{(P)} - \omega^{(A)}$ between two modes. In experiment, the change of $\omega^{(P)} - \omega^{(A)}$ is realized by adjusting the environment temperature T. The $|\omega^{(P)} - \omega^{(A)}| = 0$ is reached when the two resonances influenced by AMX have a same depth as shown in Supplementary Fig. 1, which is gotten at $T = 16.9$ °C. Under $|\omega^{(P)} - \omega^{(A)}| = 0$, the coupling rate between the two modes can be estimated by $\omega^{(s)} - \omega^{(as)} = 2K$. By fitting the transmission shown in Supplementary Fig. 1 with Eq. 1 and Eq. 2, with $\gamma^{(P)} = 0$ and $\gamma^{(A)} = 0$, we can estimate the loss factor $\alpha^{(A)}$.

Based on the coupled-mode equations, we can simulate the intracavity evolution of the dark pulses. A typical evolution process shown in Supplementary Fig. 2a can be simulated in frequency (Supplementary Fig. 2b) and time domain (Supplementary Fig. 2c) using Eq. 1 and Eq. 2 under the parameters as follows

$t_R = 10.879$ ps;

$L = 2\pi \times 144$ µm;

$\alpha^{(P)} = 0.0067$;

$\beta_2^{(P)} = 139$ ps^2 km^{-1};

$\beta_1^{(P)} = 12.280$ ns/m;

$t_R\left(\omega_0^{(A)} - \omega_0^{(P)}\right) = 0.76$;

$\theta = 0.0067$;

$\kappa = 84$ m^{-1};

$\alpha^{(A)} = 0.02$;

$\beta_2^{(A)} = 74$ ps^2 km^{-1};

$\beta_1^{(A)} = 13.438$ ns/m.

Supplementary note II: Accessibility analysis under the thermal effects

The thermal analysis in this paper is based on the model proposed in [2]. As the response time of thermal is several magnitudes slower than that of Kerr effects, the Kerr effects and thermal effects can be decoupled into two steps. In step one, the intracavity field evolution can be simulated by Eq. 1 and Eq. 2, as shown in Supplementary Fig. 3a. In step two, the thermal effects can be described by a linear model:

$$-(\omega_l-\omega_o)-\frac{\alpha_{thermal}}{K_{eff}}P_{inc}=\omega_p-\omega_l \tag{4}$$

Where $K_{eff}=\frac{n_g K_c}{\omega_o \frac{dn}{dT}}$, the ω_l is the hot cavity resonance frequency, ω_o is the cold cavity resonance frequency, ω_p is the frequency of the pump laser and n_g is the group index. The $\alpha_{thermal}$ is the thermal absorption rate, defined to be equal to $P_{thermal}/P_{inc}$, where the $P_{thermal}$ is the thermal power and the P_{inc} is the intracavity optical power. $\frac{dn}{dT}$ is the thermal-optical coefficient and K_c is the thermal conductance of the microring. In the simulation in step one, the influence of thermal effects is ignored, and the x-axis is given by $\omega_p-\omega_o$. For a given $\alpha_{thermal}$, a line, passing through the highest point on the non-mode-locked region with the slope of $k=K_{\text{eff}}/\alpha_{\text{thermal}}$, can be drawn to analyse the accessibility of the mode-locked states. If the line intersects with the mode-locked region, the intracavity state will drop to the intersection. In other words, the mode-locked region is thermal accessible.

For an evolution process of the intracavity field, the thermal tolerance can be analyzed using the line l, that passes through the highest point of non-mode-locked region and is tangent to the mode-locked region. For a larger $\alpha_{thermal}$, the whole mode-locked region will be under the line. For a smaller $\alpha_{thermal}$, part of the mode-locked region will be above the line. Thus, the line l represents the highest $\alpha_{thermal}$ under which the mode-locked region is accessible. The highest $\alpha_{thermal}$, or $\alpha_{thermal}^{max}$ for ease of expression, is selected to assess the thermal tolerance of the dark pulse generation. In experiment, the $\alpha_{thermal}$ can be extracted by fitting the resonance transmission under different pump power, and is estimated to be $0.2\alpha_i$, where the α_i is the intrinsic loss factor. The $\alpha_{thermal}^{max}$ for the dark pulse evolution processes under different pump power P_{in} is shown in Fig. 3b. The region with the $\alpha_{thermal}^{max}>0.2\alpha_i$ indicates the dark pulse state is accessible. As the $\alpha_{thermal}$ is a part of α_i, $\alpha_{thermal}$ can be normalized by α_i, which is employed in Supplementary Fig. 3b. As shown in Supplementary Fig. 3b, due to a large nonlinear coefficient, $P_{in}=0.45$

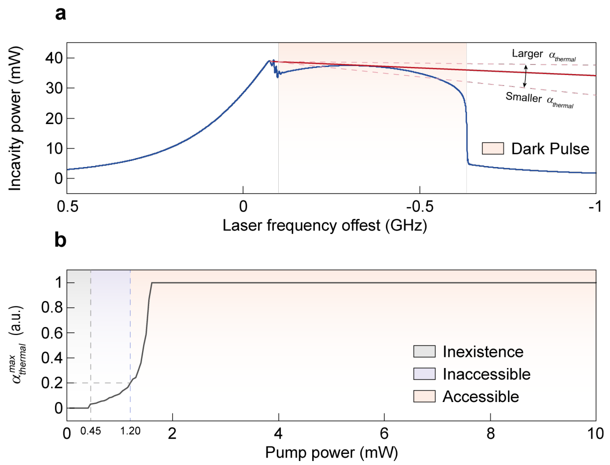

Supplementary Fig. 3: Accessibility analysis under the thermal effects. a, Simulated evolution of the average intracavity power as the pump laser tuning in a frequency-decreasing direction, employing Eq. 1 and Eq. 2. **b**, The highest thermal absorption rate $\alpha_{thermal}^{max}$, under which the dark pulses states are accessible, for different pump power.

mW is large enough to trigger dark pulses, although the large power drop blocks its stable generation. Under $P_{in} > 1.20$ mW, the dark pulse states are accessible by directly tuning the pump laser into the resonance from the long-wavelength side.

Supplementary note III: Theoretical fitting method for the RF filter responses of the MPF

The theoretical transfer function for the multi-tap delay-line microwave photonic filter (MPF) was calculated by the equation below [3, 4]:

$$H(\omega) = G(\omega) \cdot \sum_{n=0}^{N-1} p_n \exp(-jn\omega T) \tag{5}$$

where ω is the angular velocity of the input microwave signal, p_n is the optical power of the nth comb line which can be measured by the optical spectrum analyzer (OSA) during the experiment, N is the total number of the comb lines, T is the time delay unit between the adjacent comb lines.

For the MPF implemented based on non-dispersive (true-time) delay scheme, the theoretical transfer function of the MPF can be directly calculated, based on the measured p_n using Eq. 5. The parameter T is equal to the delay value of on-chip spiral delay line which is approximately 59 µs, and the $G(\omega)$ here is equal to a unit constant of 1.

For the MPF implemented based on dispersive delay line scheme, the theoretical calculation is a bit more complex, taking the third-order dispersion of the single-mode fibre (SMF) into consideration. The $G(\omega)$ here is a frequency-dependent function, which is determined by the modulation type of the high-speed electro-optical modulator (EOM). For the double sideband modulation (DSB) adopted in this work, the approximate expression of the $G(\omega)$ is shown as below [4]:

$$G(\omega) \approx \cos\left(\theta_2 \omega^2/2 + n\theta_3 \omega \Delta\omega^2/2\right) \tag{6}$$

where $\theta_2 = -\beta_2 L$ is the second-order fiber dispersion, $\theta_3 = -\beta_3 L$ is the third-order fiber dispersion, $\Delta\omega$ is the free spectral range (FSR) of the comb lines in the form of angular velocity (rad/s). T is generated by the chromatic dispersion of a spool of single-mode fibre (SMF), which is not a fixed value but as a function of the tap number n, due to the influence of the third-order dispersion. The transfer function $H(\omega)$ can be formulated as below:

$$H(\omega) = \sum_{n=0}^{N-1} G(\omega) \cdot \exp\left(jn\theta_2 \omega \Delta\omega + j\frac{1}{6}\theta_3 \omega^3 + j\frac{n^2}{2}\theta_3 \Delta\omega^2 \omega\right) \tag{7}$$

where the second-order fiber dispersion β_2 and third-order fiber dispersion β_3 are given by:

$$\beta_2 = -\frac{D\lambda^2}{2\pi c} \tag{8}$$

$$\beta_3 = \left(S - \frac{4\pi c}{\lambda^3}\beta_2\right) / \left(\frac{2\pi c}{\lambda^2}\right)^2 \tag{9}$$

The second-order dispersion parameter D and third-order dispersion parameter S of a standard SMF are ~17.4 ps/(nm·km) and ~0.083 ps/nm^2/km, respectively. Based on the Eqs. 7-9, the theoretical transfer function for arbitrary filter response can be obtained. The calculation was carried out by the numerical calculation software MATLAB.

---

[1] H. Guo, E. Lucas, M. H. P. Pfeiffer, M. Karpov, M. Anderson, J. Liu, M. Geiselmann, J. D. Jost, and T. J. Kippenberg, Intermode breather solitons in optical microresonators, Phys. Rev. X **7**, 041055 (2017).

[2] Q. Li, T. C. Briles, D. A. Westly, T. E. Drake, J. R. Stone, B. R. Ilic, S. A. Diddams, S. B. Papp, and K. Srinivasan, Stably accessing octave-spanning microresonator frequency combs in the soliton regime, Optica **4**, 193 (2017).

[3] J. Capmany, B. Ortega, and D. Pastor, A tutorial on microwave photonic filters, Journal of Lightwave Technology **24**, 201 (2006).

[4] X. Zhu, F. Chen, H. Peng, and Z. Chen, Novel programmable microwave photonic filter with arbitrary filtering shape and linear phase, Optics express **25**, 9232 (2017).
